# Drowning and the Influence of Hot Weather

**DOI:** 10.1371/journal.pone.0071689

**Published:** 2013-08-14

**Authors:** Michael Fralick, Christopher J. Denny, Donald A. Redelmeier

**Affiliations:** 1 Department of Medicine, University of Toronto, Toronto, Ontario, Canada; 2 Department of Emergency Medicine, University of Toronto, Toronto, Ontario, Canada; 3 Department of Emergency Medicine, Auckland District Health Board, Auckland, New Zealand; 4 Department of Medicine, University of Toronto, Toronto, Ontario, Canada; 5 Institute of Clinical Evaluative Sciences (ICES), Sunnybrook Health Sciences Centre, Toronto, Ontario, Canada; IUMSP, University Hospital Lausanne, Switzerland

## Abstract

**Background:**

Drowning deaths are devastating and preventable. Public perception does not regard hot weather as a common scenario for drowning deaths. The objective of our study was to test the association between hot weather and drowning risk.

**Materials and Methods:**

We conducted a retrospective case-crossover analysis of all unintentional drowning deaths in Ontario, Canada from 1999 to 2009. Demographic data were obtained from the Office of the Chief Coroner. Weather data were obtained from Environment Canada. We used the pair-matched analytic approach for the case-crossover design to contrast the weather on the date of the drowning with the weather at the same location one week prior (control period).

**Results:**

We identified 1243 drowning deaths. The mean age was 40 years, 82% were male, and most events (71%) occurred in open water. The pair-matched analytic approach indicated that temperatures exceeding 30°C were associated with a 69% increase in the risk of outdoor drowning (OR = 1.69, 95% CI 1.23–2.25, p = 0.001). For indoor drowning, however, temperatures exceeding 30°C were not associated with a statistically significant increase in the risk of drowning (OR = 1.50, 95% CI 0.53–4.21, p = 0.442). Adult men were specifically prone to drown in hot weather (OR 1.67, 95% CI 1.19–2.34, p = 0.003) yet an apparent increase in risk extended to both genders and all age groups.

**Conclusion:**

Contrary to popular belief, hot weather rather than cold stormy weather increases the risk of drowning. An awareness of this risk might encourage greater use of drowning prevention strategies known to save lives.

## Introduction

Globally, an estimated 400,000 drowning deaths occur each year. [1] In Canada, drowning is the third leading overall cause of unintentional death before the age of 60, averaging about 3 fatalities each week in Ontario (Canada’s largest province). [2] Drowning rates in Canada are similar to those of the United States, but generally lower than rates in Africa or Asia. [1] Drowning deaths are a devastating event that commonly affects young healthy individuals who might have otherwise lived a long and healthy life. Drowning deaths also carry a significant economic burden on the Canadian health care system accounting for about $106 million (Canadian dollars) in costs in 2004 [3] Despite ongoing drowning prevention strategies, the number of drowning deaths have been increasing in Canada since 2004. [4].

Most drowning deaths are preventable. [5] Ethanol use, reckless behaviour, and inadequate water safety instructions are all major modifiable risk factors. [2], [5]–[7] Personal flotation devices can save lives, as can fences surrounding swimming pools, lifeguards and swim ‘buddies’. [8]–[13] In all cases, saving lives requires that people recognize risks and avoid a false sense of security around aquatic environments. Popular media, both old and new, portray drowning events taking place during cold, stormy, dark weather. [14]–[17] Further, the Lifesaving Society of Canada as well as the Centers for Disease Control and Prevention in the United States focus on the risk of drowning during inclement weather. [18]–[19] This portrayal has the potential to create a false sense of security when people engage in aquatic activities on warm days. Few rigorous studies support the association of weather with drowning risk, and the available studies lack a control group, pertain to occupational settings, or apply only during natural disasters [20]–[22].

Most drowning deaths in Canada occur during the summer months and most occur during recreational activities. [2] We hypothesized, therefore, that hot weather rather than cool stormy weather might be associated with higher rates of drowning. To explore this hypothesis we combined data from the Office of the Chief Coroner with official governmental meteorological records to assess the association of hot weather with drowning risk. This approach used a self-matching design to control for access to health care, public health programs, population education, lifestyle patterns and multiple other confounding factors. If hot weather is associated with an increased risk of drowning, a greater awareness of this association might motivate more targeted lifesaving initiatives.

## Methods

### Data Abstraction

The study data were abstracted from the Office of the Chief Coroner of Ontario and Environment Canada. All participants in this study were deceased. The project was approved by the Sunnybrook Health Sciences REB as well as the Office of the Chief Coroner.

The next of kin did not provide their written or verbal informed consent. Neither were required nor requested by either Sunnybrook Hospital or The Office of the Chief Coroner. This is a standard operating procedure for large observational studies utilizing data from the Office of the Chief Coroner. Further, information for the next of kin was seldom available. Lastly, no identifying data was abstracted from the Coroner's Office that could plausibly identify the individual drowning victim. The Coroner Information System was secured in a locked facility and chart abstraction occurred on-site through an agreement with the Office of the Chief Coroner. The Environment Canada National Climate Data and Information Archive were obtained from the public domain.

### Study Setting

We conducted a retrospective case-crossover analysis of drowning victims in Ontario from January 1, 1999 to January 1, 2009. Ontario had a population of 12,160,280 in 2009 and a land area of over one million square kilometres. [23] Ontario has approximately 250,000 lakes, four of which make up the largest continuous body of fresh water in the world denoted as The Great Lakes. [24] Over 98% of the Ontario population lives within the drainage catchment basin of The Great Lakes. [24].

### Inclusion and Exclusion Criteria

**Figure 1:**
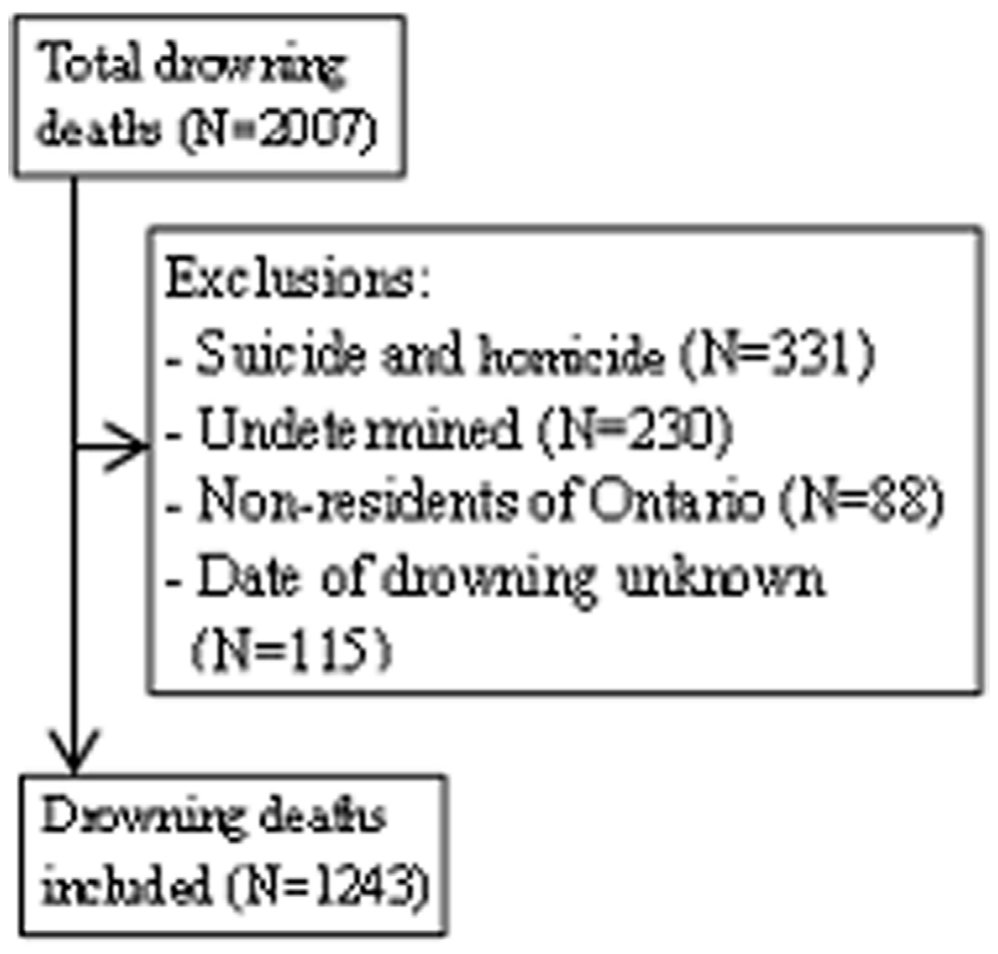
Drowning deaths in the province of Ontario (1999–2009).

Using Coroner’s records, we identified consecutive deaths among Ontario residents aged 1 day and older during the ten year study interval. The study interval up until 2009 was selected because it was the most recent decade available that provided adequate sample size taking into account the lag time for Coroner’s reports. We excluded non-fatal drowning, intentional events (eg, suicide or homicide), deaths among non-residents of Ontario, and cases where the date of drowning was unknown (Figure 1). [25] Non-fatal drowning deaths were excluded since the data were not available at the Office of the Chief Coroner or any other centralized database. Non-residents of Ontario were excluded due to the lack of identifying information necessary for demographic data and medical comorbidities.The date and time of the drowning was determined from the police report where available and the coroner’s report otherwise.

### Individual Characteristics

Data on individual characteristics were obtained from the Coroner’s charts including demographic information, location, cause of drowning, drug toxicology reports, and co-morbid medical conditions known to potentially increase drowning risk (eg, epilepsy). These evaluations combined information from both the regional coroner and police forces. We analyzed indoor drowning deaths as a pre-specified secondary analysis since we did not believe indoor drowning deaths (eg, bathtub, indoor pools) would be influenced by the weather. The relationship between age and gender and drowning risk was analyzed as post-hoc analyses. No other post-hoc analyses were conducted. The datasets did not include information on swimming skill, medications, or recent illness.

### Meteorological Data

All weather data were obtained from Environment Canada’s National Climate Data and Information Archive. [26] Ontario has approximately 389 weather stations (average of 1 station per 2500 square kilometers). When hourly data were available, we identified the hour nearest the drowning death for weather measurements. We used daily maximum temperature in cases where data were infrequent or when the time of the drowning was imprecise. The weather station in the town or city of the drowning was used when available. If no such station was available, we used the weather station in the municipality or, failing that, the nearest neighbouring weather station. We selected air temperature as a consistent element of weather because it was measured objectively, routinely captured, and easily interpreted by the lay-population in daily activities.

### Statistical Analysis

We applied the case-crossover design, a technique for assessing transient fluctuations such as weather on the risk of an acute event such as drowning. [27] This is a self-matching population-based design that controlled for multiple confounders including public safety campaigns, seasonal recreational activities, local alcohol sales, and population demographics. Applying the case-crossover design allows each case to serve as his or her own control, thereby avoiding confounding due to age, gender, swimming ability, chronic health conditions (eg, epilepsy), and other fixed characteristics.

Our statistical analysis used the pair-matched approach, based on McNemar’s test to contrast the weather on the date of the drowning (hazard interval) with the weather at the same location at the same time one week before the drowning (control interval). The same weather station was used for both comparison intervals. A seven-day separation between the two intervals was used to control for weekday, season, and year. Analyses were applied using univariate regression based on the full dataset and all p-values were two-tailed.

## Results

### Drowning Cases

We identified 1243 drowning deaths in Ontario during the ten year study, equivalent to about 120 deaths per year for a population of about 12 million people. About 82% were male (n = 1020). The mean age was 40 years and about one quarter were over the age of 55 years (Table 1). The majority occurred in outdoor locations and the majority had not been wearing a personal flotation device. About one in three had been drinking alcohol. Cardiac disease was the most common predisposing medical condition., although most cases had no medical comorbidity (Table 2).

**Table 1:** Drowning Characteristics.

	N = 1243	Count (%)
Gender	Male	1020 (82)
	Female	223 (18)
Age	0–17y	216 (17)
	18–24y	156 (13)
	25–39y	245 (20)
	40–54y	290 (23)
	55–69y	171 (14)
	>69y	165 (13)
Location	Outdoor	1072 (86)
	Indoor*	171 (14)
Personal flotation device (PFD)	PFD not present	996 (80)
	PFD present, not worn	138 (11)
	PFD worn	37 (3)
	Not specified	72 (6)

* Indoor drowning deaths included both bathtub drownings (N = 135) and indoor pool drownings.

**Table 2:** Location of drowning and medical comorbidities.

	N = 1243	Count (%)
Location	Open water	876 (71)
	Bath tub	135 (11)
	Private pool	103 (8)
	Pond or quarry	61 (5)
	Public pool	27 (2)
	Unspecified	40 (3)
Co-morbidity*	Cardiac	83 (7)
	Respiratory	11 (1)
	Epilepsy	74 (6)
	Diabetes	24 (2)
	Psychiatric	58 (5)
	Drug intoxication	54 (4)
	Neurologic	35 (3)
	Trauma	35 (3)
	None specified	859 (69)

* Up to two comorbid conditions were recorded per drowning.

### Primary Analysis

Overall, 130 of the drowning days (10%) had a temperature exceeding 30°C at the location of the drowning (hazard interval). In contrast, 85 of the control days (7%) had a temperature exceeding 30°C during the same time at the same location one week earlier (control interval). In total, 18 of the days (1%) had a temperature exceeding 30°C at the same location during both the drowning day and the control day. The pair-matched analytic approach indicated that daily air temperatures exceeding 30°C were associated with a 69% increase in the risk of drowning (odds-ratio = 1.69, 95% confidence interval 1.23 to 2.25, p = 0.001). The increase risk was mostly explained by individuals without cardiac disease (OR = 1.60, 95% CI 1.18 to 2.19, p = 0.003) and only partially explained by individuals with cardiac disease (OR = 2.71, 95% CI 0.80 to 9.24, p = 0.08).

### Secondary Test of Robustness

We checked our work with a pre-specified secondary analysis restricted to indoor drowning deaths (n = 171). Overall, 10 cases (6%) had a temperature exceeding 30°C at the location of the drowning, 7 cases (4%) had a temperature exceeding 30°C during the same time at the same location one week earlier, and 1 case (1%) had a temperature exceeding 30°C at the same location during both the drowning day and the control day. The pair-matched analytic approach indicated that temperatures exceeding 30°C were associated with a non statistically significant increase in drowning risk (odds-ratio = 1.50, 95% confidence interval 0.53 to 4.21, p = 0.442). Notably, the lack of statistical significance may be a reflection of the relatively small sample size of indoor drowning deaths.

We found consistent results after assessing the impact of age and gender upon the risk of death by outdoor drowning. The increase in drowning risk associated with hot weather was apparent for adults (odds-ratio 1.61, 95% confidence interval 1.12 to 2.3, p = 0.009) and had marginal statistical significance for children (odds-ratio 2.00, 95% confidence interval 1.00 to 3.99, p = 0.050). Rechecking the association by redefining childhood as below 18 years of age, rather than less than 12 years of age, yielded a significant association for adults (OR 1.59, 95% confidence interval 1.13–2.20, p = 0.007) and for children (OR = 2.00, 95% confidence interval, 1.03–3.89, p = 0.041). Males were specifically prone to drown in hot weather (odds-ratio = 1.67, 95% confidence interval 1.19–2.34, p 0.003) whereas females showed an increase in risk that was similar in magnitude but not statistically significant (odds-ratio = 1.86, 95% confidence interval 0.74–8.60, p = 0.187).

## Discussion

Our study is the first to analyze the association between hot weather and drowning risk for over one thousand deaths during ten years in Canada. We found that the number of outdoor drownings increased significantly on days when the maximum temperature exceeds 30°C. Men were specifically prone to drown on hot days compared to cool days. Children were twice as likely to drown on hot days compared to cool days. These findings are unlikely to reflect random chance, differences in water safety knowledge, or the availability of protective equipment. Instead, the data suggest that hot weather rather than cold stormy weather increases the risk of death by drowning.

Previous case series of drowning deaths in the fishing industry and drowning deaths following natural disasters emphasize the hazards of stormy weather. [20]–[22] We do not doubt this risk is real, but highlight that such risk reflects a minority of drowning deaths in Canada. Data from large case series in China and Bangladesh found that drowning deaths increased with temperature elevations. [28]–[29] Our study has formally tested this hypothesis in a four-season climate and our results confirm that hot weather is the greater danger to public health. Further, a recent report from the Office of the Chief Coroner supports our findings that hot weather is a common element in drowning risk [30].

Several reasons might explain why hot temperatures are associated with higher rates of drowning. First, drowning is directly correlated with aquatic exposure. [28], [31]–[32] As temperature rises, therefore, people spend more time around the water. Second, alcohol use is a risk factor for drowning and consumption may increase on hot weather days. [2], [32]–[33] Alcohol negatively affects judgment and swimming ability, perhaps explaining the distinct drowning risk for adult men since men are more likely to be under the influence of alcohol during aquatic activities and engage in risky behaviours. [33] In addition, use of a personal flotation device may decline on warm summer days among all age groups. Although some studies have demonstrated that cardiac mortality rates increase at the extremes of temperature, [34] these studies excluded accidental deaths (e.g., drowning) and focus on the elderly or patients with multiple medical comorbidities. [35]–[36] Since most deaths in our study were young adults with no medical combordities it is unlikely that the observed increased drowning rates are related to underlying trends in cardiac mortality related to hot weather.

Several weaknesses of this study merit emphasis. First, our study design was retrospective and cannot determine causality; in particular, we do not know whether individuals were involved in water-related activities during the control day and thus the observed increased risk may be due to increased aquatic exposure alone. Second, we selected a single control period for study feasibility, whereas additional control periods might have improved the robustness of our findings. Third, we attempted to record hourly weather data when possible, yet hourly meteorological data were not available for all drowning deaths. Since the weather can vary within a given day, we cannot be certain that daily maximum temperature accurately reflected the air temperature during the individual drowning (a second source of measure error that also slants our analysis to the null). Fourth, our data set did not include non-fatal drowning events. [25] Since non-fatal drowning events occur approximately five times more often than drowning deaths, our study substantially underestimates the absolute risks [1], [5].

Some additional limitations of our study also merit consideration. Our study occurred in a high-income country with universal access to health care; hence, our results may not be applicable to low income countries where drowning rates are particularly high. We were not able to control for long-weekends marked by holidays; however, such imbalances may be partially offset by drowning deaths that occurred one week after a long-weekend, since the long-weekend would now become the control interval. We also did not have access to data regarding drowning risk factors from the control interval (e.g., alcohol intake). Also, air temperature may not be the most accurate measurement for “weather”. Instead, cloud coverage, humidity, wind speed, or rainfall might represent further distinctions that influences a person’s behaviour in water-related activities. Studying the effect of water temperature on drowning risk would also be important, but was not feasible for this study as the data is not available from Environment Canada. Our analysis was not powered to analyze multiple air temperature thresholds. Lastly, although indoor drowning deaths were not associated with hot weather, this may be due to a small sample size of indoor drownings and this lack of an association does not necessarily confirm our study hypothesis. Further research is justified to address these limitations.

Our study suggests an association but not necessarily a causal relationship between hot weather and the risk of drowning. For example, hot weather may lead to increased water-related activities, increased alcohol consumption, increased reckless behaviour, or lower use of a personal flotation device. [22] In effect, hot weather alone may not increase drowning risk but may influence behavioural changes that predispose to drowning. These mechanisms, therefore, provide opportunities for diverse drowning prevention initiatives. None of these uncertainties justify a nihilistic approach to water safety.

Strategies for drowning prevention fall into three categories; namely, education, engineering, and enforcement. [37] Although our study does not address the effectiveness of these strategies they are fundamentally important to prevent future drowning deaths. More persons could be educated about the risk of drowning associated with hot weather and, in particular, receive specific warnings about responsible alcohol consumption during hot weather days. A lighter, sleeker inflatable personal flotation device or other technologic improvements might also help to prevent future drowning deaths. Applying stricter legislation for personal flotation device use is another option, as is continued enforcement of alcohol-free watercraft operation. An awareness of risk can lead to thoughtful action whereas a popular misconception can result in misdirected resources and misfortunate consequences.
